# Molecular mechanism of m^6^A methylation of circDLC1 mediated by RNA methyltransferase METTL3 in the malignant proliferation of glioma cells

**DOI:** 10.1038/s41420-022-00979-6

**Published:** 2022-04-26

**Authors:** Quansheng Wu, Xiaofeng Yin, Wenbo Zhao, Wenli Xu, Laizhao Chen

**Affiliations:** Department of neurosurgery, The Second Hospital of Shanxi Medical University, 030001 Taiyuan City, Shanxi Province China

**Keywords:** Tissue engineering, Experimental models of disease

## Abstract

Glioma is an intracranial malignant tumor and remains largely incurable. Circular RNAs are prominent modulators in glioma progression. This study investigated the function of circular RNA DLC1 (circDLC1) in the malignant proliferation of glioma cells. circDLC1 expression in glioma tissues and cells was determined using RT-qPCR. The effect of circDLC1 on the malignant proliferation of glioma cells was analyzed using CCK-8, colony formation, and EdU staining assays. METTL3, miR-671-5p, and CTNNBIP1 expressions were determined. N^6^ methyladenosine (m^6^A) level of circDLC1 was analyzed using MeRIP. The binding relationship between miR-671-5p and circDLC1 or CTNNBIP1 was verified using RNA pull-down and dual-luciferase assays. A xenograft tumor model was established in nude mice to verify the effect of METTL3-mediated circDLC1 on glioma in vivo. circDLC1 was poorly expressed in glioma. circDLC1 overexpression suppressed glioma cell proliferation. Mechanically, METTL3-mediated m^6^A modification enhanced circDLC1 stability and upregulated circDLC1 expression in glioma. circDLC1 upregulated CTNNBIP1 transcription by competitively binding to miR-671-5p. METTL3 overexpression repressed the malignant proliferation of glioma via circDLC1/miR-671-5p/CTNNBIP1 in vivo. Collectively, METTL3-mediated m^6^A modification upregulated circDLC1 expression, and circDLC1 promoted CTNNBIP1 transcription by sponging miR-671-5p, thus repressing the malignant proliferation of glioma.

## Introduction

Glioma is a heterogeneous group of primary malignancies of the central nervous system [1], which is notorious for its rapid cell proliferation and angiogenesis, and particularly the tumors cells are resistant to conventional treatments, resulting in poor outcomes and a high incidence of relapse [2, 3]. Glioma patients experience dismal outcomes due to the deficient knowledge of molecular pathogenesis and the lack of effective diagnostic and therapeutic strategies [4]. Consequently, investigations on the molecular mechanism of glioma malignant proliferation have aroused extensive concerns.

Circular RNAs (circRNAs) are a novel class of covalently linked single-stranded RNAs with a closed loop structure [5]. circRNAs are abundantly expressed in the brain tissues, indicating their critical roles in various brain diseases [6]. circRNAs exhibit differential expressions in glioma and modulate glioma progression [7]. circDLC1 is derived from exons 14, 15, and 16 of the DLC1 gene, and circDLC1 overexpression can repress the proliferation of hepatoma cells [8]. Nevertheless, the exact role of circDLC1 in glioma remains unknown.

Recently, N^6^-methyladenosine (m^6^A) modification is emphasized for its abundant enrichment and critical biological functions in circRNAs [9]. m^6^A modification emerges as methylation at the N^6^ position of adenosine, representing the most prevalent internal modification in eukaryotes mRNA [10]. The aberrant alternation of m^6^A level affects RNA maturation, transcription, translation, and metabolism, which disrupts gene expression and vital cellular processes, and ultimately leads to the initiation and progression of multiple tumors, including glioma [11]. Methyltransferase-like 3 (METTL3) is one of the “writers” for m^6^A methylation that participate in regulating various cellular biological processes [12]. METTL3 is poorly expressed in glioma tissues, and METTL3 deficiency facilitates glioma cell proliferation [13]. METTL3 is implicated in the biogenesis and function of circRNAs by mediating m^6^A modification [9]. However, the mechanism of METTL3-mediated m^6^A modification on circDLC1 in glioma has not been investigated before.

Mechanistically, circRNAs can modulate the expression of genes by serving as a competitive endogenous RNA (ceRNA) of microRNA (miRNA) [14]. miRNAs are a class of small endogenous non-coding RNAs (18–25 nt in length) [15]. miRNA affects the functions of genes involving gliomagenesis, tumor growth, therapeutic response, and prognosis, which is a prospective therapeutic tool against malignant glioma [16]. This study probed into the effect of METTL3-mediated m^6^A methylation of circDLC1 on the malignant proliferation of glioma cells and the downstream ceRNA mechanism of circDLC1, hoping to shed light on glioma treatment.

## Results

### circDLC1 was poorly expressed in glioma tissues and cells

**Fig. 1:**
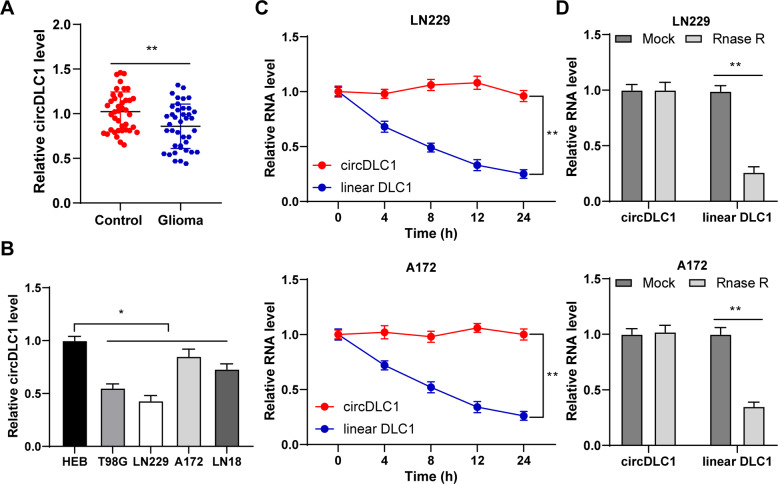
circDLC1 was poorly expressed in glioma. **A** circDLC1 expression in glioma tissues and adjacent tissues was determined using RT-qPCR. **B** circDLC1 expression in human glioma cell lines and normal glioma cell line was detected using RT-qPCR. **C**, **D** circDLC1 and linear DLC1 in cells were detected using RT-qPCR after actinomycin D or RNase R treatment. *N* = 40. The cell experiment was repeated 3 times independently. Data in panels **B**–**D** are presented as mean ± standard deviation. Data in panel **A** were analyzed using paired *t*-test. Data in panel **B** were analyzed using one-way ANOVA, and data in panels **C**, **D** were analyzed using two-way ANOVA, followed by Tukey’s multiple comparisons test, *p < 0.05, **p < 0.01.

circDLC1 is poorly expressed in hepatocellular carcinoma [8], but its role in glioma is unclear. Our results exhibited that circDLC1 expression was reduced in glioma tissues (*p* < 0.01, Fig. 1A). Also, circDLC1 was poorly expressed in glioma cells (*p* < 0.01, Fig. 1B). circDLC1 level had no significant change after actinomycin D or RNase R treatment, but linear DLC1 level was decreased notably (*p* < 0.01, Fig. 1C), indicating that circDLC1 was more stable than linear DLC1 (*p* < 0.01, Fig. 1D). All these demonstrated that circDLC1 was poorly expressed in glioma tissues and cells.

### Overexpression of circDLC1 suppressed malignant proliferation of glioma cells

**Fig. 2:**
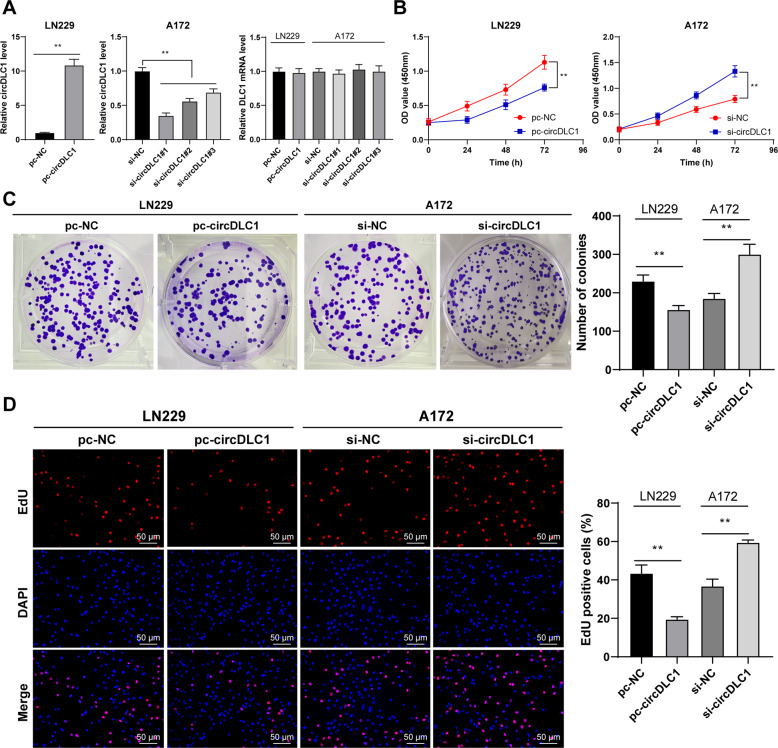
circDLC1 overexpression suppressed glioma cell proliferation. circDLC1 pcDNA 3.1 (pc-circDLC1) was transfected into LN229 cells, with NC pcDNA 3.1 (pc-NC) as control; circDLC1 siRNA (si-circDLC1) was transfected into A172 cells, with NC siRNA (si-NC) as control. **A** circDLC1 and linear DLC1 expressions were detected using RT-qPCR. **B**–**D** Cell proliferation was measured using CCK-8 assay (**B**), colony formation assay (**C**), and EdU staining (**D**). The cell experiment was repeated three times independently. Data are presented as mean ± standard deviation. The comparisons between two groups in panels **A**/C, **D** were analyzed using *t*-test. The comparisons among multiple groups in panel **A** were analyzed using one-way ANOVA, and the comparisons among multiple groups in panel **B** were analyzed using two-way ANOVA, followed by Tukey’s multiple comparisons test, **p < 0.01.

To explore the effect of circDLC1 on the malignant proliferation of glioma cells, we transfected circDLC1 pcDNA 3.1 (pc-circDLC1) into LN229 cells with relatively low circDLC1 expression (*p* < 0.01, Fig. 2A) and tansfected circDLC1 siRNA (si-circDLC1) into A172 cells with relatively high circDLC1 expression (*p* < 0.01, Fig. 2A). si-circDLC1#1 with the best intervention efficiency was selected for subsequent detection. Overexpression or silencing of circDLC1 did not affect the expression of linear DLC1 (Fig. 2A). circDLC1 overexpression decreased glioma cell proliferation and circDLC1 silencing enhanced glioma cell proliferation (*p* < 0.01, Fig. 2B–D). Briefly, overexpression of circDLC1 suppressed the malignant proliferation of glioma cells.

### METTL3-mediated m^6^A modification enhances circDLC1 stability and upregulates circDLC1 expression

**Fig. 3:**
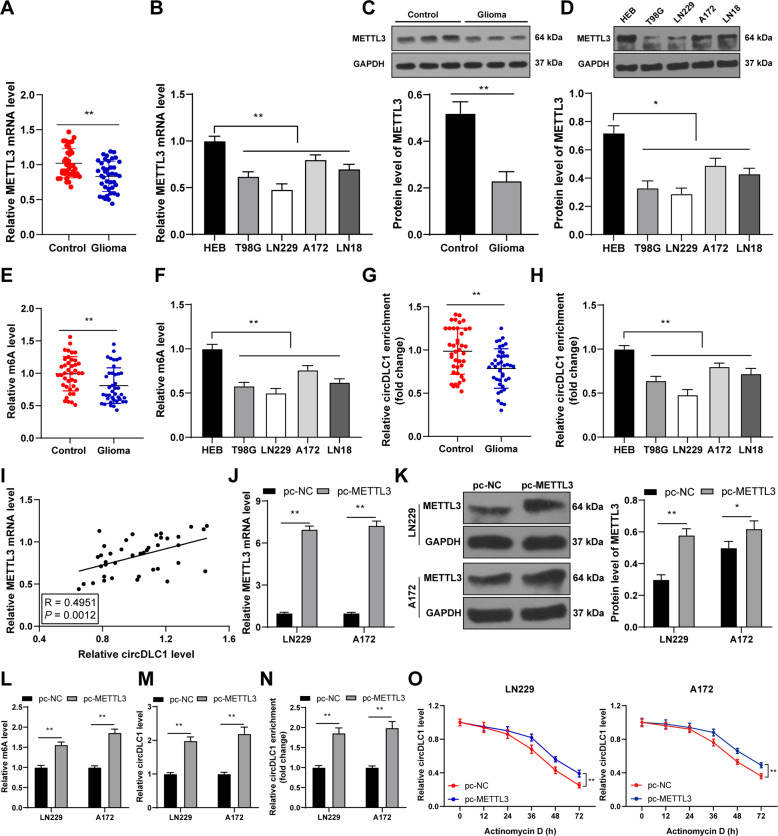
METTL3-mediated m^6^A modification enhanced circDLC1 stability and upregulated circDLC1 expression. **A**–**D** METTL3 expression in glioma tissues and cells was detected using RT-qPCR and western blot. **E**, **F** m^6^A quantitative analysis of m^6^A level in tumor tissues and cells. **G**, **H** MeRIP analysis of m^6^A level of circDLC1 in tumor tissues and cells. **I** Pearson correlation analysis of the correlation between METTL3 and circDLC1 in 40 cases of glioma. METTL3 pcDNA 3.1 (pc-METTL3) was transfected into LN229 and A172 cells, respectively, with NC pcDNA 3.1 (pc-NC) as NC. **J**, **K** METTL3 expression in cells was detected using RT-qPCR and western blot. **L** m^6^A quantitative analysis of m^6^A level in cells. **M** circDLC1 expression in cells was detected using RT-qPCR. **N** MeRIP analysis of m^6^A level of circDLC1 in cells. **O** After actinomycin D treatment, circDLC1 expression in cells was detected using RT-qPCR. *N* = 40. The cell experiment was repeated three times independently. Data in panels **B**–**D**/**F**/**H**/**J**–**O** are presented as mean ± standard deviation. The comparisons between two groups in panels **A**/**E**/**G** were analyzed using paired *t*-test, and the comparisons between two groups in panels **C**/**J**–**N** were analyzed using *t*-test. The comparisons among multiple groups in panels **B**/**D**/**F**/**H** were analyzed using one-way ANOVA, and the comparisons among multiple groups in panel **O** were analyzed using two-way ANOVA, followed by Tukey’s multiple comparisons test, *p < 0.05, **p < 0.01.

m^6^A modification exists in circDLC1 [8]. METTL3, as an m^6^A modifying enzyme, is poorly expressed in glioma [13, 17]. Our results exhibited that METTL3 expression was reduced in glioma tissues and cells (*p* < 0.05, Fig. 3A–D). The m^6^A level in glioma tissues and cells was consistent with the trend of METTL3 expression (*p* < 0.01, Fig. 3E–F). The m^6^A level of circDLC1 was decreased in glioma tissues and cells (*p* < 0.01, Fig. 3G–H). METTL3 expression was positively correlated with circDLC1 expression in glioma tissues (*p* < 0.01, Fig. 3I). After METTL3 pcDNA 3.1 (pc-METTL3) was transfected into glioma cells (*p* < 0.05, Fig. 3J–K), the m^6^A level in cells, circDLC1 expression, and m^6^A level of circDLC1 were also increased (*p* < 0.01, Fig. 3L–N). The half-life of circDLC1 was prolonged after METTL3 overexpression (*p* < 0.01, Fig. 3O). Briefly, METTL3-mediated m^6^A modification upregulated circDLC1 expression by increasing circDLC1 stability.

### Overexpression of METTL3 annulled the promoting effect of circDLC1 silencing on the malignant proliferation of glioma cells

**Fig. 4:**
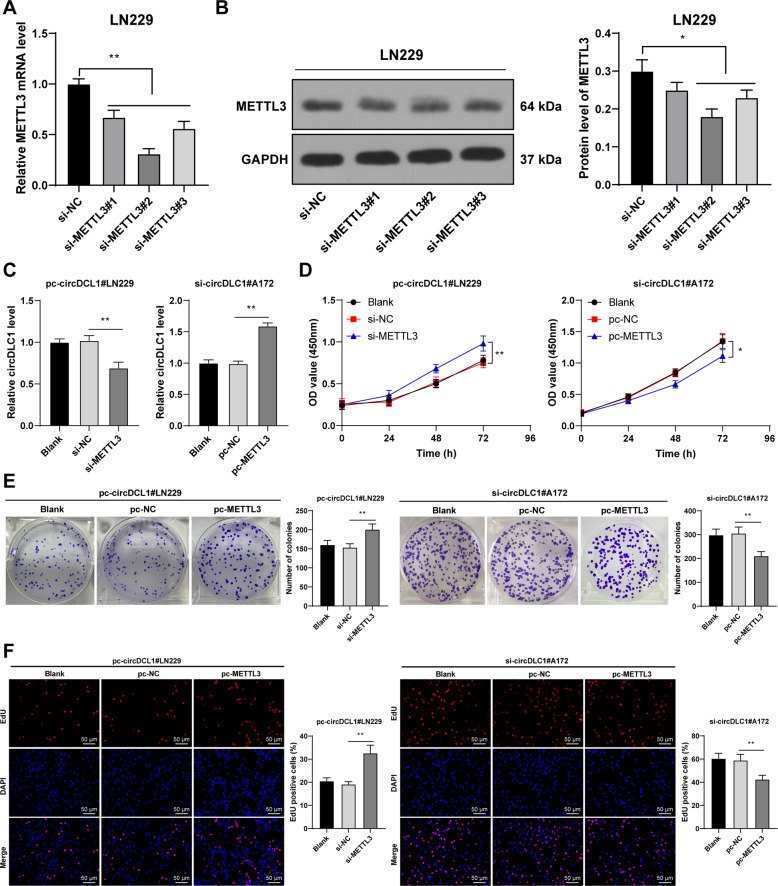
Overexpression of METTL3 annulled the promoting effect of circDLC1 silencing on glioma cell malignant proliferation. A172 cells were treated with METTL3 overexpression and circDLC1 silencing. METTL3 siRNA (si-METTL3) was transfected into LN229 cells, with NC siRNA (si-NC) as control. **A**, **B** METTL3 expression in cells was detected using RT-qPCR (**A**) and western blot (**B**). **C** circDLC1 expression of cells in the combined treatment group was detected using RT-qPCR. **D**–**F** Cell proliferation was measured using CCK-8 assay (**D**), colony formation assay (**E**), and EdU staining (**F**). The cell experiment was repeated three times independently. Data are presented as mean ± standard deviation. Data in panels **A**–**C**/**E**, **F** were analyzed using one-way ANOVA, and data in panel **D** were analyzed using two-way ANOVA, followed by Tukey’s multiple comparisons test, *p < 0.05, **p < 0.01.

To verify the role of METTL3 in the regulation of glioma cells by circDLC1, we transfected three METTL3 siRNAs (si-METTL3) into LN229 cells respectively (*p* < 0.05, Fig. 4A, B) and selected si-METTL3#2 with the best transfection efficiency for subsequent experiments. Compared with circDLC1 overexpression alone, circDLC1 overexpression + METTL3 silencing enhanced LN229 cell proliferation; compared with circDLC1 silencing alone, METTL3 overexpression + circDLC1 silencing increased circDLC1 expression in A172 cells and decreased A172 cell proliferation (*p* < 0.05, Fig. 4C–F). Briefly, METTL3 overexpression annulled the promoting effect of circDLC1 silencing on glioma cell proliferation.

### circDLC1 promoted CTNNBIP1 transcription by competitively binding to miR-671-5p

**Fig. 5:**
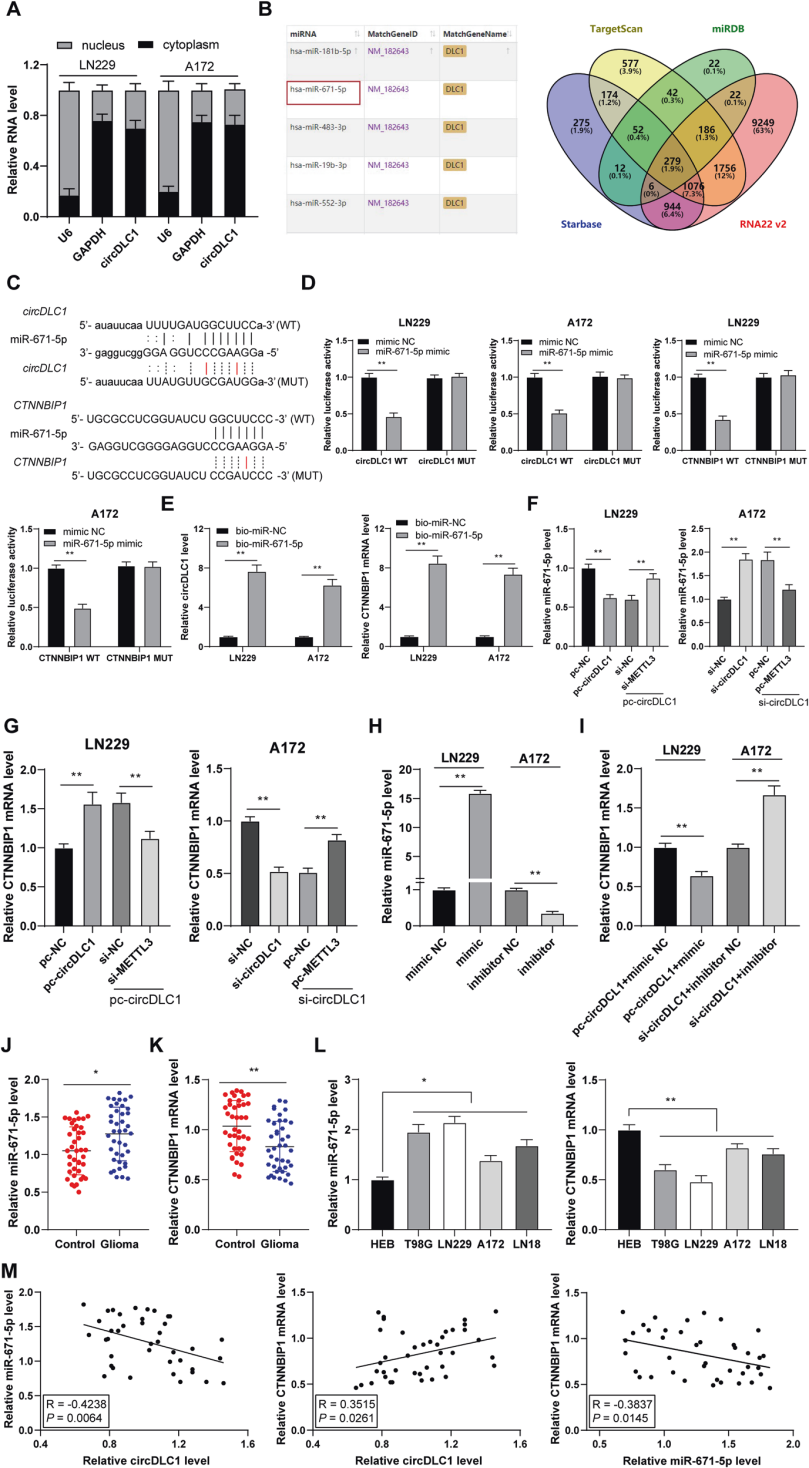
circDLC1 promoted CTNNBIP1 transcription by competitively binding to miR-671-5p. **A** The location of circDLC1 in LN229 and A172 cells was analyzed using nuclear/cytosol fractionation assay. **B** The miRNAs downstream of circDLC1 were predicted (partial results), or the downstream genes of miR-671-5p were predicted through the Starbase, Targetscan, RNA22 v2, and miRDB databases. **C** The binding sites of miR-671-5p with circDLC1 or CTNNBIP1 in the Starbase and Targetscan databases. **D**, **E** The binding relationship between miR-671-5p and circDLC1 or CTNNBIP1 was verified using dual-luciferase and RNA pull-down assays. **F**, **G** miR-671-5p and CTNNBIP1 expressions in cells were determined using RT-qPCR; miR-671-5p mimic or inhibitor was transfected into glioma cells, with NC mimic or inhibitor as control; the transfected cells were treated with pc-circDLC1 or si-circDLC1. **H**, **I** miR-671-5p and CTNNBIP1 expressions in cells were determined using RT-qPCR. **J**–**L** miR-671-5p and CTNNBIP1 expressions in glioma tissues and cells were determined using RT-qPCR. **M** Pearson correlation analysis of the correlation among miR-671-5p, CTNNBIP1, and circDLC1 in 40 cases of glioma tissues. *N* = 40. The cell experiment was repeated three times independently. Data in panels **A**/**D**–**I**/**L** are presented as mean ± standard deviation. The comparisons between two groups in panels **J, K** were analyzed using paired *t*-test. The comparisons among multiple groups in panels **F**–**I**/**L** were analyzed using one-way ANOVA, and the comparisons among multiple groups in panels **D**, **E** were analyzed using two-way ANOVA, followed by Tukey’s multiple comparisons test, *p < 0.05, **p < 0.01.

Next, we explored the downstream mechanism of circDLC1. circDLC1 was mainly localized in the cytoplasm of glioma cells (Fig. 5A), suggesting that circDLC1 may bind to miRNA [18]. The downstream miRNAs of circDLC1 were predicted (Supplementary Table 2 and Fig. 5B), in which miR-671-5p is highly expressed in glioma [19, 20]. The target genes of miR-671-5p were predicted and intersected (Fig. 5B), in which CTNNBIP1 is poorly expressed in glioma [21, 22]. We identified the binding sites between circDLC1 and miR-671-5p, and miR-671-5p and CTNNBIP1 through the database (Fig. 5C). Dual-luciferase and RNA pull-down assays confirmed the binding relationships between circDLC1 and miR-671-5p, and miR-671-5p and CTNNBIP1 (*p* < 0.01, Fig. 5D, E). circDLC1 overexpression alone decreased miR-671-5p expression and circDLC1 silencing alone increased miR-671-5p expression, while METTL3 silencing or METTL3 overexpression reduced the regulatory effect of intervention of circDLC1 on miR-671-5p expression; the transcriptional level trend of CTNNBIP1 was opposite to that of miR-671-5p (p < 0.01, Fig. 5F, G). Then, we intervened in miR-671-5p expression (p < 0.01, Fig. 5H) and found that miR-671-5p overexpression decreased the CTNNBIP1 transcriptional level in the combined treatment group, while miR-671-5p silencing increased the CTNNBIP1 transcriptional level (*p* < 0.01, Fig. 5I). Finally, we found that miR-671-5p expression was elevated and CTNNBIP1 expression was reduced in glioma tissues and cells (*p* < 0.05, Fig. 5J–L). miR-671-5p expression was negatively correlated with circDLC1 and CTNNBIP1 expression in glioma tissues, and CTNNBIP1 expression was positively correlated with circDLC1 expression (*p* < 0.05, Fig. 5M). Briefly, circDLC1 promoted CTNNBIP1 transcription by competitively binding to miR-671-5p.

### CTNNBIP1 silencing attenuated the inhibitory effect of circDLC1 overexpression on malignant proliferation of glioma cells

**Fig. 6:**
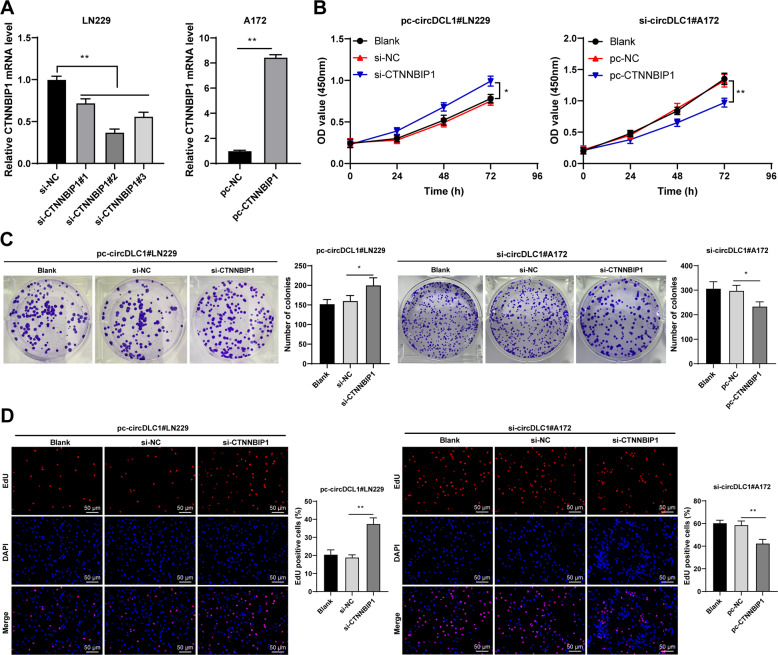
CTNNBIP1 silencing attenuated the inhibitory effect of circDLC1 overexpression on malignant proliferation of glioma cells. Three CTNNBIP1 siRNAs (si-CTNNBIP1) were transfected into LN229 cells, with NC siRNA (si-NC) as control. CTNNBIP1 pcDNA 3.1 (pc-CTNNBIP1) was transfected into A172 cells, with NC pcDNA 3.1 (pc-NC) as control. **A** CTNNBIP1 mRNA expression was determined using RT-qPCR. **B**–**D** Cell proliferation was measured using CCK-8 assay (**B**), colony formation assay (**C**), and EdU staining (**D**). The cell experiment was repeated three times independently. Data are presented as mean ± standard deviation. The comparisons between two groups in panel **A** were analyzed using *t*-test. The comparisons among multiple groups in panels **A**/**C**, **D** were performed using one-way ANOVA, and the comparisons among multiple groups in panel **B** were analyzed using two-way ANOVA, followed by Tukey’s multiple comparisons test, *p < 0.05, **p < 0.01.

Then, we verified the regulatory effect of CTNNBIP1 on glioma cells. After three CTNNBIP1 siRNAs (si-CTNNBIP1) were transfected into LN229 cells, CTNNBIP1 mRNA expression in LN229 cells was decreased (*p* < 0.01, Fig. 6A). We chose si-CTNNBIP1#2 and pc-circDLC1 to treat LN229 cells. Also, we transfected CTNNBIP1 pcDNA 3.1 (pc-CTNNBIP1) into A172 cells (p < 0.01, Fig. 6A), and then, pc-CTNNBIP1-treated A172 cells were transfected with si-CTNNBIP1. si-CTNNBIP1#2 + pc-circDLC1 promoted LN229 cell proliferation, while pc-CTNNBIP1 + si-circDLC1 inhibited A172 cell proliferation (*p* < 0.05, Fig. 6B–D). Briefly, CTNNBIP1 silencing attenuated the inhibitory effect of circDLC1 overexpression on glioma cell proliferation.

### METTL3 suppressed glioma cell proliferation via the circDLC1/miR-671-5p/CTNNBIP1 axis in vivo

**Fig. 7:**
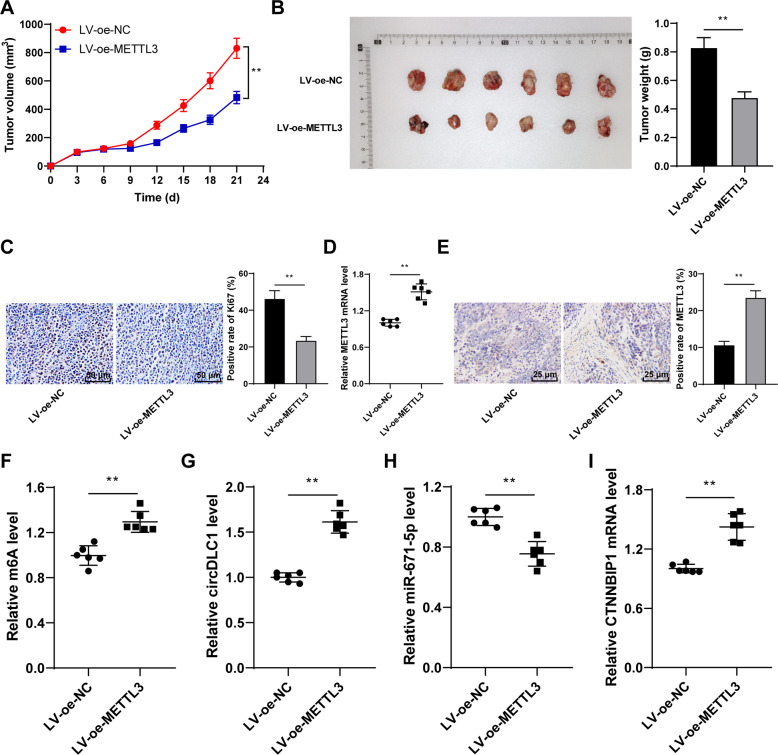
METTL3 suppressed glioma cell proliferation via the circDLC1/miR-671-5p/CTNNBIP1 axis in vivo. LN229 cells stably overexpressing METTL3 (LV-oe-METTL3) were used to establish the xenograft tumor model. **A** Tumor volume. **B** Images of stripped tumors and weight of tumors after the nude mice were euthanized on the 21st day. **C** Ki67-positive rate was detected using immunohistochemistry. **D**, **E** METTL3 expression was determined using RT-qPCR and immunohistochemistry. **F** m^6^A quantitative analysis of m^6^A level in tumor tissues. **G**–**I** circDLC1, miR-671-5p, and CTNNBIP1 expressions were determined using RT-qPCR. *N* = 6. The cell experiment was repeated three times independently. Data in panels **A–C**/**E** are presented as mean ± standard deviation. Data in panels **B**–**I** were analyzed using *t*-test. Data in panel **A** were analyzed using two-way ANOVA, followed by Tukey’s multiple comparisons test, **p < 0.01.

Finally, we verified the regulation of METTL3-mediated circDLC1 on glioma cells in vivo. METTL3 overexpression suppressed tumor growth (*p* < 0.01, Fig. 7A–C). Compared with the LV-oe-NC group, the LV-oe-METTL3 group showed increased METTL3 expression (*p* < 0.01, Fig. 7D–E), elevated m6A level and circDLC1 expression (*p* < 0.01, Fig. 7F–G), reduced miR-671-5p expression (*p* < 0.01, Fig. 7H), and enhanced CTNNBIP1 expression (*p* < 0.01, Fig. 7I). Briefly, METTL3 suppressed glioma cell malignant proliferation via the circDLC1/miR-671-5p/CTNNBIP1 axis in vivo.

## Discussion

**Fig. 8:**
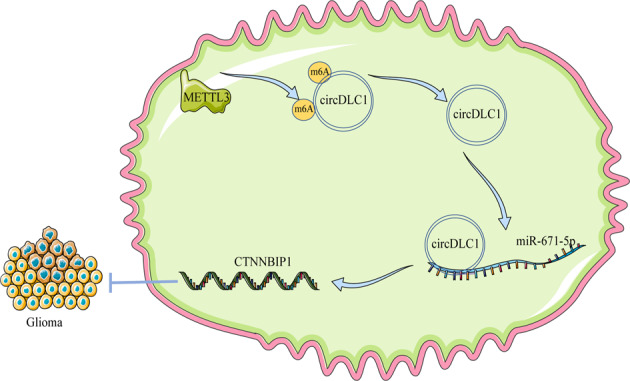
Molecular mechanism of circDLC1 regulating malignant proliferation of glioma cells. METTL3-mediated m^6^A modification can increase the stability of circDLC1, upregulate the expression of circDLC1 in glioma cells, and promote the competitive binding of circDLC1 and miR-671-5p, thereby upregulating the transcription of CTNNBIP1 and then inhibiting the malignant proliferation of glioma cells.

Glioma presents a challenging clinical situation that causes notable mortality and morbidity [1]. circRNAs regulate cell cycle, angiogenesis, metastasis, and drug resistance of glioma cells [23]. This study elucidated that METTL3-mediated circDLC1 promoted CTNNBIP1 transcription by sponging miR-671-5p, thus repressing the malignant proliferation of glioma cells (Fig. 8).

Patients with lower circDLC1 expression exhibit a more advanced hepatocellular carcinoma stage [8]. Nevertheless, the role of circDLC1 in glioma remained unknown. In the present study, we found that circDLC1 was poorly expressed in glioma tissues and cells. Unlike linear RNAs, the circular structure of circRNAs contributes to their stability in tissues and ensures their capacity to participate in various biological events [24]. Actinomycin D or RNase R treatment did not change circDLC1 level but reduced linear DLC1 level significantly, indicating that circDLC1 was more stable than linear DLC1. Then, we intervened in circDLC1 expression in glioma cells and found that circDLC1 overexpression suppressed glioma cell proliferation. We were the first to demonstrate that circDLC1 expression was reduced in glioma, and circDLC1 overexpression suppressed the malignant proliferation of glioma cells.

The biogenesis and functions of circRNAs can be mediated by m^6^A modification [25]. circDLC1 is regulated by m^6^A modification in hepatocellular carcinoma pathogenesis [8]. The reduction of m^6^A level in glioma can be partially attributed to the decreased METTL3 level [17]. METTL3 is a well-studied RNA methyltransferase that executes an m^6^A-dependent modification of circRNAs implicated in carcinogenesis [26, 27]. Our results exhibited that the m^6^A level, METTL3 expression, and m^6^A level of circDLC1 were reduced in glioma tissues and cells. METTL3 expression showed a positive correlation with circDLC1 in glioma tissues. Consensus m^6^A motifs are abundant in circRNAs and METTL3 enhances m^6^A-driven RNA translation [28]. We demonstrated that METTL3-mediated m^6^A modification increased circDLC1 expression by enhancing circDLC1 stability. METTL3 downregulation underlies a potential mechanism of glioma tumorigenesis [13]. METTL3 knockdown dramatically facilitates glioblastoma stem cell growth, self-renewal, and tumor progression [29]. Similarly, we showed that METTL3 overexpression annulled the promoting effect of circDLC1 silencing on glioma cell proliferation.

circRNAs modulate gene expression by functioning as miRNA sponges [14]. miR-671-5p is encoded by a gene localized at 7q36.1, which is amplified in glioblastoma multiforme [20]. miR-671-5p is highly expressed in glioblastoma cells and facilitates the proliferation and reduces the apoptosis of glioblastoma cells [21]. CTNNBIP1 serves as a tumor suppressor and is demonstrated to be diminished in glioma [22]. We demonstrated that miR-671-5p expression was elevated and CTNNBIP1 expression was declined in glioma tissues and cells, and circDLC1 promoted CTNNBIP1 transcription by binding to miR-671-5p. CTNNBIP1 downregulation is related to unfavorable prognosis, high histological grade, and advanced glioma progression [21]. Consistently, we found that CTNNBIP1 silencing attenuated the inhibitory effect of circDLC1 overexpression on glioma cell proliferation. Finally, we verified the regulatory mechanism of METTL3-mediated circDLC1 in vivo. The reduction of METTL3-mediated m^6^A modification results in the in vivo growth of glioma cells [13]. Our results exhibited that METTL3 overexpression repressed tumor growth, elevated m^6^A level and circDLC1 expression, reduced miR-671-5p expression, and enhanced CTNNBIP1 expression. Briefly, METTL3 suppressed the glioma cell proliferation via the circDLC1/miR-671-5p/CTNNBIP1 axis in vivo.

To sum up, METTL3-mediated m^6^A modification upregulated circDLC1 expression by enhancing the stability of circDLC1, thereby promoting the competitive binding of circDLC1 and miR-671-5p, facilitating the transcription of CTNNBIP1, and eventually repressing the malignant proliferation of glioma cells. This study first reported circDLC1 expression patterns in glioma and elucidated its effect on the malignant proliferation of glioma cells. However, this study only explored the role of the miR-671-5p/CTNNBIP1 axis downstream of circDLC1, and there are still many miRNAs downstream of circDLC1 to be explored. Whether circDLC1 is also affected by other m^6^A modifying enzymes needs to be further studied. In addition, we only detected the effect of the change of CTNNBIP1 transcription level on the malignant proliferation of glioma cells, and the effect of the change of CTNNBIP1 protein level is not clear. In the future, we will verify the effect of CTNNBIP1 protein level on glioma cell proliferation and explore other possible mechanisms of circDLC1.

## Materials and methods

### Ethics statement

This study was approved by the Ethical Committee of The Second Hospital of Shanxi Medical University and in accordance with *Helsinki Declaration*. The animal experiments were approved by the Experimental Animal Welfare and Ethics Committee of The Second Hospital of Shanxi Medical University. All patients had signed the written informed consent. All the animal experiments were implemented based on the *Guide for the Care and Use of Laboratory Animals* [30].

### Clinical sample collection

We collected the tumor tissues of 40 patients (at an average age of 54.35 ± 9.52; 21 males and 19 females) with primary glioma and corresponding adjacent tissues in The Second Hospital of Shanxi Medical University from June 2016 to June 2018 and stored in liquid nitrogen at –80 °C. None patients received chemotherapy or radiotherapy before. The histological features of collected tissues were independently diagnosed by two pathologists.

### Cell culture

Human glioma cell lines (T98G, LN229, A172, and LN18) and healthy glioma cell line (HEB) were obtained from American Type Culture Collection (Manassas, Virginia, USA). All cells were subjected to short tandem repeat (STR) identification and cultured in RPMI-1640 medium (HyClone, South Logan, UT, USA) containing 10% heat-inactivated fetal bovine serum (Invitrogen, Carlsbad, CA, USA), 100 U/mL penicillin, and 100 μg/mL streptomycin (Thermo Fisher Scientific, Waltham, MA, USA) at 37 °C with 5% CO_2_.

### Cell treatment

Three siRNAs specifically targeting circDLC1, circDLC1 pcDNA 3.1, three siRNAs specifically targeting METTL3, METTL3 pcDNA 3.1, three siRNAs specifically targeting catenin beta interacting protein 1 (CTNNBIP1), CTNNBIP1 pcDNA 3.1, miR-671-5p mimic, miR-671-5p inhibitor, and their negative controls (NCs) were designed and synthesized by GenePharma (Shanghai, China). Finally, these vectors were transfected into A172 or LN229 cells using Lipofectamine 3000 (Thermo Fisher Scientific).

### Cell-counting kit-8 (CCK-8) assay

A172 and LN229 cells were seeded into 96-well plates (2000 cells/well) and cultured for 0, 24, 48, and 72 h. Each well was added with 10 μL CCK-8 reagent (Beyotime, Beijing, China) for 2-h incubation at 37 °C. The absorbance at the wavelength of 450 nm was measured using a microplate reader (Bio-Rad, Philadelphia, PA, USA).

### Colony formation assay

A172 or LN229 cells (200–300 cells) were seeded into the petri dish in humidified air at 37 °C with 5% CO_2_ for 10 days. After the removal of the complete medium, the cells were fixed with 4% paraformaldehyde and stained with 0.1% crystal violet for 20 min. Colony cells were counted under an optical microscope (Nikon, Tokyo, Japan).

### 5-ethynyl-2ʹ-deoxyuridine (EdU) staining

A172 and LN229 cells were seeded into 96-well plates (20,000 cells) and cultured in the medium (100 μL/well, ThermoFisher) containing 50 μM EdU for 2 h (5% CO_2_, 37 °C). Briefly, the cells were treated with 4% paraformaldehyde for 30 min and stained with 100 μL Apollo dye solution (Ribobio) for 30 min. The nuclei were stained with DAPI (ThermoFisher) for 30 min, followed by observation under the fluorescence microscope (Olympus, Tokyo, Japan). The final results are presented as a percentage.

### Actinomycin D treatment

A172 or LN229 cells were seeded into 24-well plates (5 × 10^5^ cells/well). After 24 h, the cells were treated with 1 μg/mL actinomycin D. The relative RNA expressions of circDLC1 and DLC1 were analyzed using reverse transcription quantitative polymerase chain reaction (RT-qPCR).

### RNase R treatment assay

The total RNA of A172 and LN229 cells (3 μg) was incubated with 10 U RNase R (20 U/μL, Epicentre, Madison, WI, USA) at 37 °C for 30 min and at 75 °C for 10 min to deactivate the RNase R, followed by RT-qPCR.

### m^6^A level detection

The m6A level in total RNA was measured using m6A RNA methylation quantitative kit (ab185912, Abcam Inc., Cambridge, MA, USA). Firstly, RNA was coated in the detection well at 37 °C for 90 min, then added with capture antibody (50 μL), detection antibody (50 μL), and enhancer solution (50 μL) respectively, and incubated at room temperature for 60 min. Finally, the chromogenic solution was added for signal detection and absorbance measurement. The m^6^A level was measured using the colorimetry method (450 nm).

### m^6^A RNA immunoprecipitation (MeRIP) assay

MeRIP was performed using Magna MeRIP m6A kit (Millipore, Billerica, MA, USA) to detect the m6A level of circDLC1. Briefly, 3 μg anti-m6A antibody (ab208577, Abcam) was incubated with protein A/G magnetic beads at 4 °C overnight. Then, the conjugate was incubated with RNA in the IP buffer containing RNase inhibitor and protease inhibitor. RNA was isolated and detected using RT-qPCR.

### Nuclear/cytosol fractionation assay

After rinsing with pre-cooled phosphate-buffered saline (PBS), 1 × 10^6^ A172 or LN229 cells were resuspended in 1 mL RLN buffer (50 mM Tris-HCl pH 7.4, 0.14 M NaCl, 1.5 mM MgCl_2_, 0.5% IGEPAL CA-630, and 1 mM DTT), incubated on ice for 5 min, and then homogenized. The cytoplasm was the supernatant after centrifugation. The precipitate was resuspended in 1 mL RSB buffer, homogenized, and centrifuged to remove the residual cytoplasm. The preliminary nucleus was resuspended in 3 mL 2 M RSB (2 M sucrose, 10 mM Tris-HCl pH 7.4, 10 mM NaCl, 3 mM MgCl_2_, 1 mM DTT, and 0.5 mM PMSF) and centrifuged at 3000 × *g* for 45 min. The isolated circDLC1 was subjected to RT-qPCR analysis, with U6 and glyceraldehyde 3-phosphate dehydrogenase (GAPDH) as nuclear and cytoplasmic controls, respectively [31].

### Dual-luciferase assay

Wild-type (WT) circDLC1 or CTNNBIP1 containing miR-671-5p binding sequence was synthesized and inserted into the pmirGLO vector (Promega, Madison, WI, USA) and named circDLC1 WT or CTNNBIP1 WT. Mutant-type (MUT) circDLC1 or CTNNBIP1 containing miR-671-5p mutant binding sequence was synthesized and inserted into the pmirGLO vector, named circDLC1 MUT or CTNNBIP1 MUT. The constructed plasmids were co-transfected with miR-671-5p mimic or mimic NC into A172 and LN229 cells using Lipofectamine 3000, respectively. The luciferase activity was measured after 48 h using a luciferase detection kit (Promega).

### RNA pull-down assay

miR-671-5p and miR-NC were biotinylated using the biotin RNA Labeling Mix (Roche, Indianapolis, IN, USA) and transcribed using T7/SP6 RN polymerase (Roche). Then, biotin-labeled miR-671-5p (bio-miR-671-5p) and bio-miR-NC were treated with RNase free DNase I (Promega) and RNeasy Mini kit (Qiagen, Redwood City, CA, USA). Next, M-280 streptavidin magnetic beads were incubated with bio-miR-671-5p or bio-miR-NC at room temperature for 4 h, and then with A172 and LN229 cell lysates. The RNA binding to bio-miR-671-5p or bio-miR-NC was extracted with TRIzol reagent for RT-qPCR.

### Xenograft tumor experiments

Male BALB/c nude mice (aged 6 weeks old; weighing 20 g) purchased from Vital River Laboratory Animal Technology Co., Ltd (Beijing, China) [SYXK (Beijing) 2017-0033] were raised in a standard animal room and maintained under a 12-h light/dark cycle, with food and water provided ad libitum. LN229 cells were infected with the lentiviral overexpression vector of METTL3 (LV-oe-METTL3) or NC (LV-oe-NC) obtained from Genechem (Shanghai, China). Afterward, puromycin was used to screen the stably expressed cells. Before the experiment, the nude mice were weighed and numbered. The researchers divided them into two groups using the random number method. LN229 cells (5 × 10^5^) were injected into the posterior flank of mice subcutaneously and tumor growth was observed. The health status of mice was detected every 3 days and the the tumor size was measured. The tumor volume was calculated: volume (mm^3^) = (length × width^2^)/2. The mice were sacrificed by intraperitoneal injection of 1% pentobarbital sodium (150 mg/kg) 21 days after injection. The tumors were removed and weighed. During the experiment, the researchers tried their best to reduce the number of nude mice and the pain of nude mice, and there was no animal death in the whole process. To ensure the reliability of experimental data, the tumor tissues of 6 mice in each group were fixed with 4% paraformaldehyde and embedded in paraffin for immunohistochemistry. The tumor tissues of the remaining six mice were used to detect the expression of cytokines in the tumor homogenate using RT-qPCR.

### Immunohistochemistry

The tumor tissue of mice was fixed with 4% paraformaldehyde, embedded in paraffin, and cut into 4 μm sections. After dewaxing and dehydration, the sections were incubated with METTL3 antibody (ab195352, Abcam) and Ki67 antibody (ab15580, Abcam) at 4 °C overnight. After PBS washing, the sections were incubated with the secondary antibody (ab6721, Abcam) at 37 °C for 20 min. After 2,4-diaminobutyric acid development (10 min) and hematoxylin counterstaining (3 min), the sections were dehydrated with gradient alcohol, cleared with xylene, and sealed with neutral resin. The sections were observed by professionals under a microscope using a double-blind method. Five visual fields were randomly selected from the section, and the positive rate of each visual field was calculated [32].

### Reverse transcription quantitative polymerase chain reaction (RT-qPCR)

Total RNA was extracted using the TRIzol reagent (Invitrogen) and 2 μg RNA was reverse transcribed into cDNA using SuperScript RT kit (Invitrogen). RT-qPCR was performed using SYBR Premix ExTaq^TM^ (Takara, Kyoto, Japan) and Applied Biosystems 7300 system (Applied Biosystems, Inc., Carlsbad, CA, USA). PCR primers are shown in Supplementary Table 1. The relative expression was calculated using the 2^–ΔΔCt^ method [33], with GAPDH and U6 as internal references [34].

### Western blot

Total protein was extracted using radio-immunoprecipitation assay buffer. The extract was centrifuged at 4 °C and 12,000 × *g* for 15 min and the protein concentration was determined using bicinchoninic acid kits (Beyotime). The protein was separated using 10% sodium dodecyl sulfate-polyacrylamide gel electrophoresis (SDS-PAGE) and transferred onto polyvinylidene fluoride membranes (Millipore). The membranes were blocked with 5% skim milk for 1 h and incubated with the primary antibodies METTL3 (ab195352, 1:1000, Abcam) and GAPDH (ab9485, 1:2000, Abcam) at 4 °C overnight. Following tris-buffered saline-tween buffer washing, the membranes were incubated with the secondary antibody (ab6721, 1:2000, Abcam) at 37 °C for 1 h. NIH Image J (National Institutes of Health, Bethesda, Maryland, USA) was used to analyze the gray value, with GAPDH as internal reference.

### Bioinformatics analysis

The downstream miRNAs of circDLC1 were predicted through the Starbase database (http://starbase.sysu.edu.cn/index.php) [35]. The downstream genes of miR-671-5p were predicted through the Starbase database, Targetscan database (http://www.targetscan.org/vert_71/) [36], RNA22 v2 database (https://cm.jefferson.edu/rna22/Precomputed/) [37], and miRDB database (http://mirdb.org/index.html) [38].

### Statistical analysis

Data analysis and map plotting were performed using the SPSS 21.0 (IBM Corp., Armonk, NY, USA) and GraphPad Prism 8.0 (GraphPad Software Inc., San Diego, CA, USA). The data complied with the assumption of normality and homogeneity of variance. Data are expressed as mean ± standard deviation. The *t*-test was adopted for comparisons between two groups. One-way or two-way analysis of variance (ANOVA) was employed for the comparisons among multiple groups, following Tukey’s multiple comparison test. A value of *p* < 0.01 indicated a significant difference.

## Supplementary information

Original western blots

Supplementary Table 1

Supplementary Table 2

## Data Availability

The datasets used and/or analyzed during the current study are available from the corresponding author on reasonable request.
