# Supplementary material for: Molecular mechanism of m6A methylation of circDLC1 mediated by RNA methyltransferase METTL3 in the malignant proliferation of glioma cells
Source: Cell Death Discov. 2022 Apr 26;8:229. doi: 10.1038/s41420-022-00979-6 (PMC9043209; doi:10.1038/s41420-022-00979-6)

**Figure 3C**

METTL3 and GAPDH


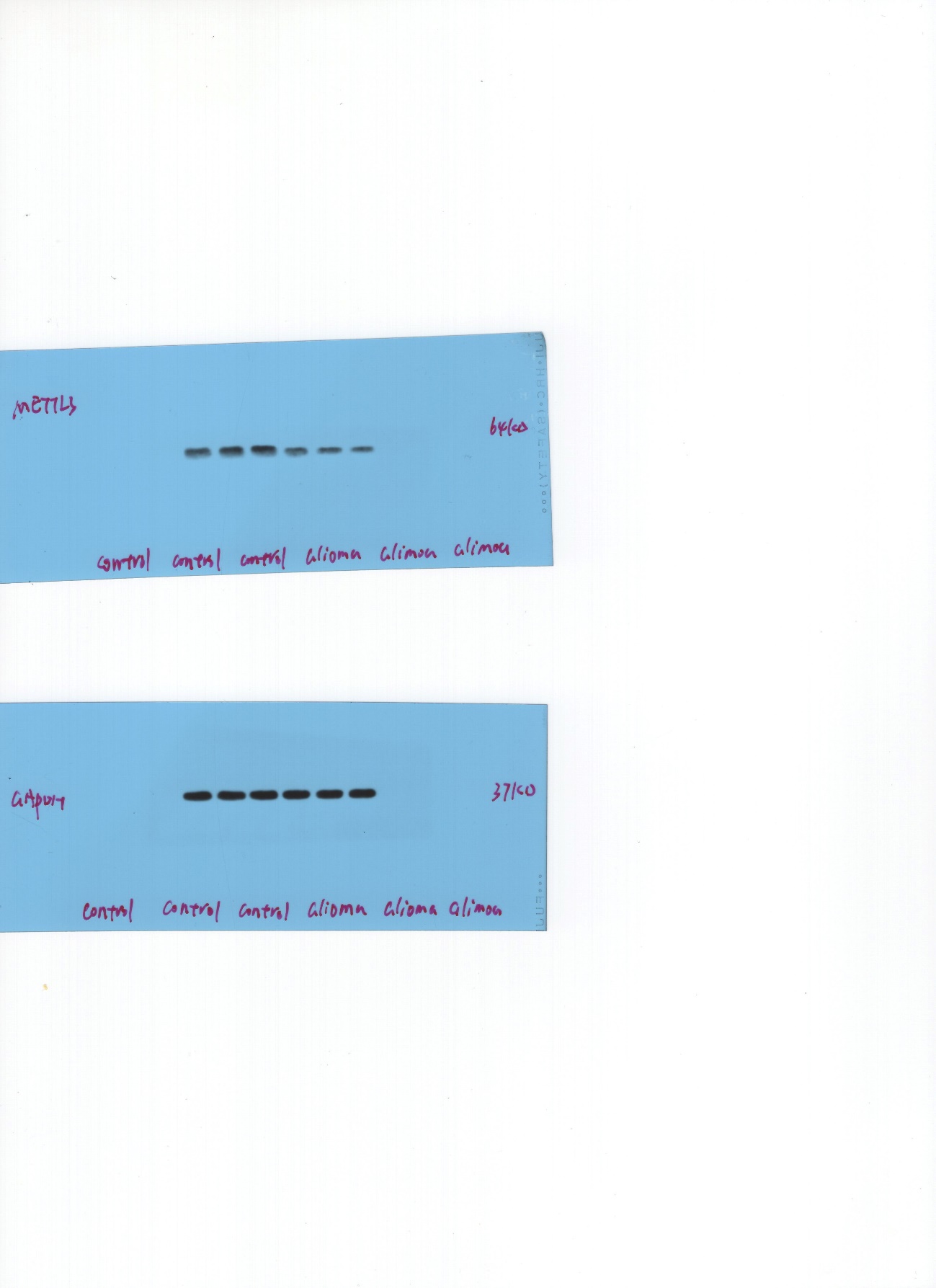


**Figure 3D**

METTL3 and GAPDH


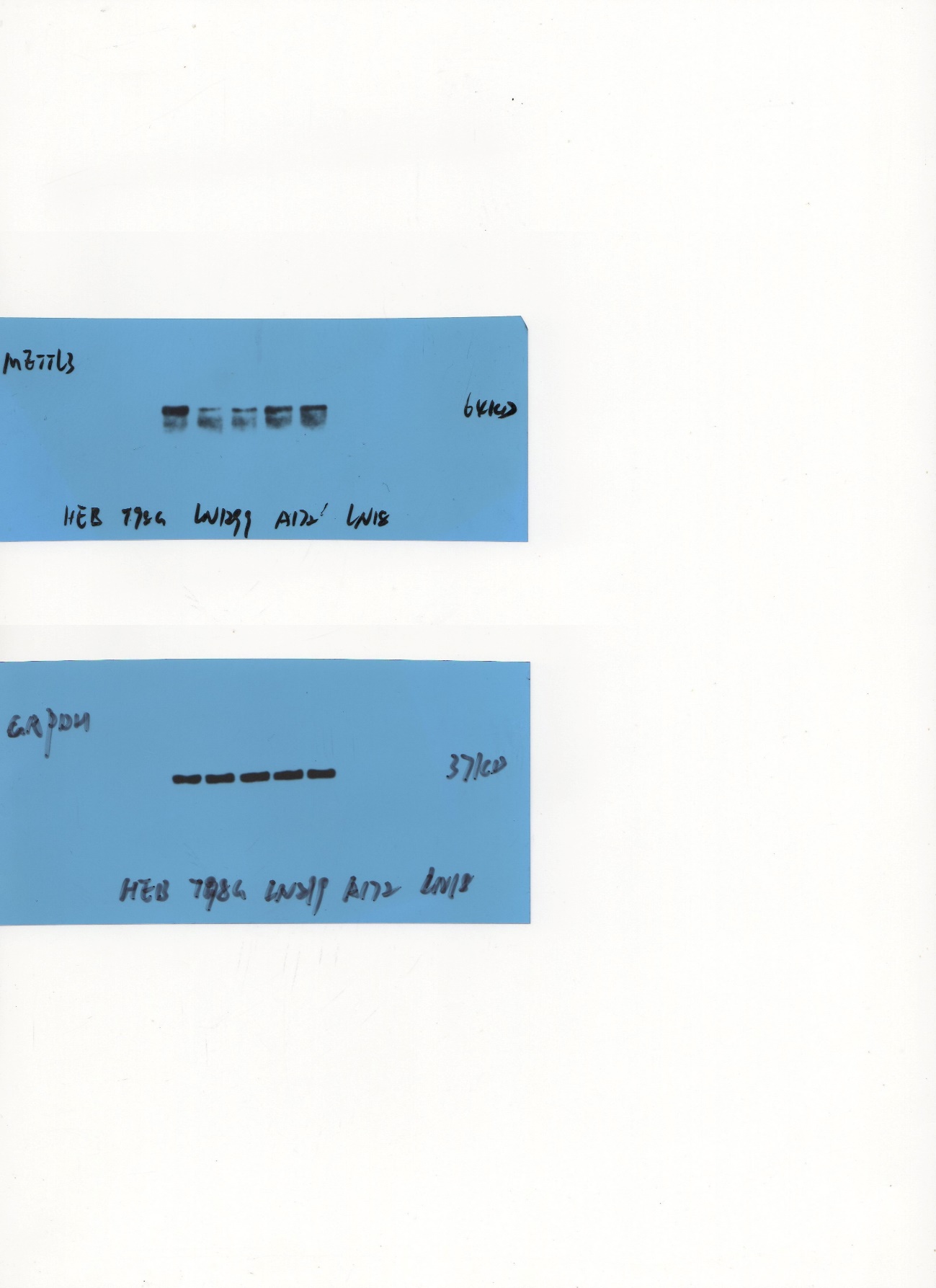


**Figure 3K**

LN229: METTL3 and GAPDH


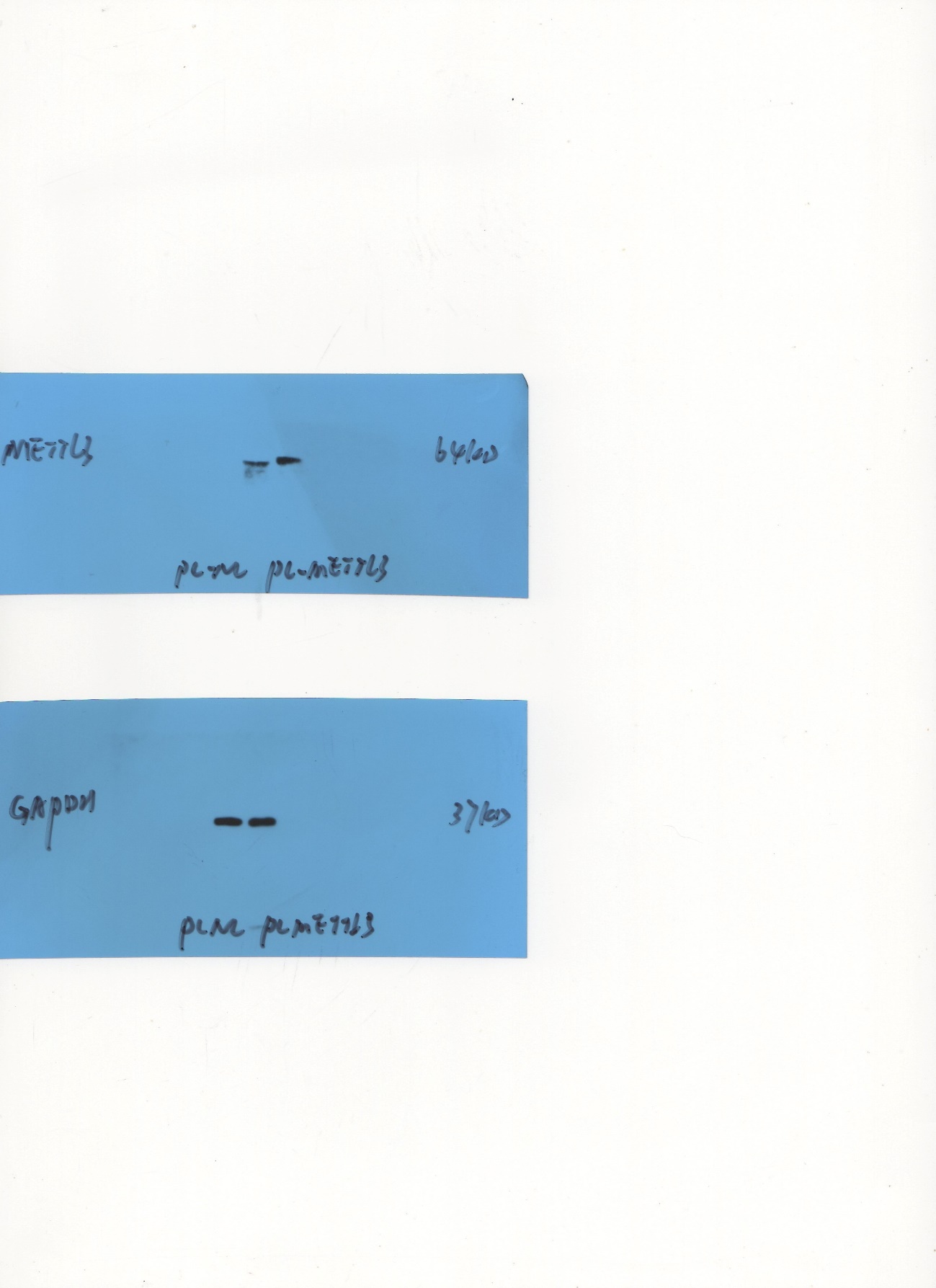


**Figure 3K**

A172: METTL3 and GAPDH


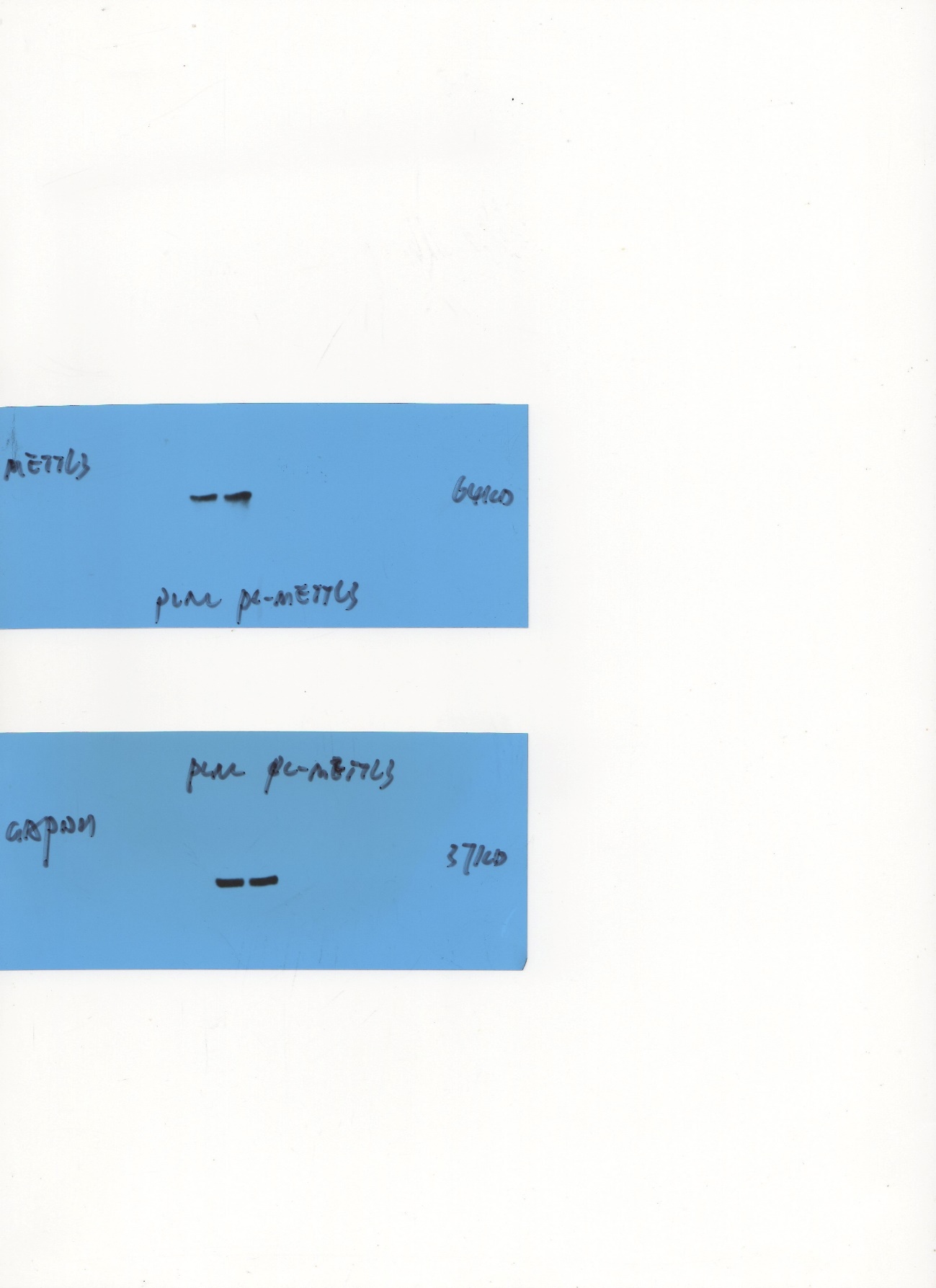


**Figure 4B**

METTL3 and GAPDH


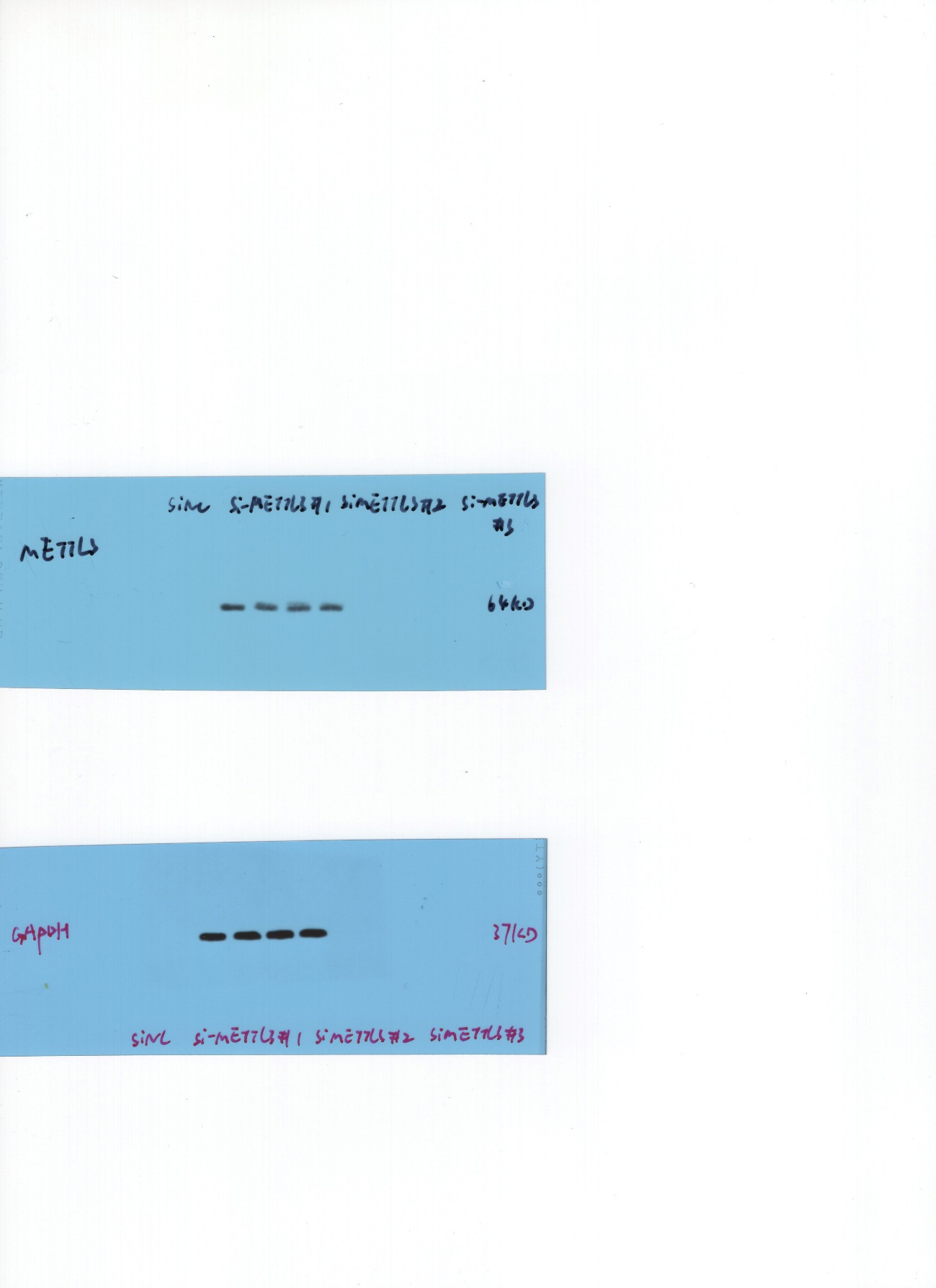

Supplement: Supplementary file 1 — Original western blots [file 41420_2022_979_MOESM1_ESM.docx]
