# Supplementary material for: Molecular mechanism of m6A methylation of circDLC1 mediated by RNA methyltransferase METTL3 in the malignant proliferation of glioma cells
Source: Cell Death Discov. 2022 Apr 26;8:229. doi: 10.1038/s41420-022-00979-6 (PMC9043209; doi:10.1038/s41420-022-00979-6)
Supplement: Supplementary file 2 — Supplementary Table 1 [file 41420_2022_979_MOESM2_ESM.docx]

**Supplementary Table 1 PCR primer sequences**

| Name | Sequences (5’-3’) |
| --- | --- |
| circDLC1 | F: TATGGTGAGGGTCATGAAGGA |
|  | R: ACCAAATTACGAAGTGGTCGC |
| METTL3 | F: TGGGGGTATGAACGGGTAGA |
|  | R: TGGTTGAAGCCTTGGGGATT |
| DLC1 | F: AGCTTTTCCAGGTTCCCGAG |
|  | R: AGGGGCTTCAGCTCTTGTTC |
| miR-671-5p | F: ATGAGTAGGAAGCCCTGGAG |
|  | R: TCAACTGGTGTCGTGGAGTC |
| CTNNBIP1 | F: TATGCAGGGGTGGTCAACAG |
|  | R: GACCTGGAAAACGCCATCAC |
| GAPDH | F: ATGGTTTACATGTTCCAATATGA |
|  | R: TTACTCCTTGGAGGCCATGTGG |
| U6 | F: TCGCTTCGGCAGCACATATACT |
|  | R: GCTTCACGAATTTGCGTGTCATC |

**Note:** circDLC1: circular RNA DLC1; METTL3: methyltransferase-like 3; miR-671-5p: microRNA-671-5p; CTNNBIP1: catenin beta interacting protein 1; GAPDH: glyceraldehyde 3-phosphate dehydrogenase
